# Cough and sputum in long COVID are associated with severe acute COVID-19: a Japanese cohort study

**DOI:** 10.1186/s12931-023-02591-3

**Published:** 2023-11-14

**Authors:** Mayuko Watase, Jun Miyata, Hideki Terai, Keeya Sunata, Emiko Matsuyama, Takanori Asakura, Ho Namkoong, Katsunori Masaki, Kazuma Yagi, Keiko Ohgino, Shotaro Chubachi, Ichiro Kawada, Takao Mochimaru, Ryosuke Satomi, Yoshitaka Oyamada, Keigo Kobayashi, Toshiyuki Hirano, Takashi Inoue, Ho Lee, Kai Sugihara, Nao Omori, Koichi Sayama, Shuko Mashimo, Yasushi Makino, Tatsuya Kaido, Makoto Ishii, Koichi Fukunaga

**Affiliations:** 1 Division of Pulmonary Medicine, Department of Medicine, Keio University School of Medicine, 35 Shinanomachi, Shinjuku-ku, Tokyo, 160-8582 Japan; 2 Keio Cancer Center, Keio University School of Medicine, 35 Shinanomachi, Shinjuku, Tokyo, 160-8582 Tokyo Japan; 3 Department of Infectious Diseases, Keio University School of Medicine, Tokyo, Japan; 4 Keio University Health Center, Tokyo, Japan; 5 Department of Respiratory Medicine, National Hospital Organization Tokyo Medical Center, Tokyo, Japan; 6 Department of Internal Medicine, Sano Kosei General Hospital, Tochigi, Japan; 7 Department of Pulmonary Medicine, Kawasaki Municipal Hospital, Kawasaki, Kanagawa Japan; 8 Department of Respiratory Medicine, Toyohashi Municipal Hospital, Toyohashi, Aichi Japan; 9 Department of Respiratory Medicine, Nagoya University Graduate School of Medicine, Nagoya, Japan

**Keywords:** Cough, Japanese cohort study, Long COVID, Severity, Sputum

## Abstract

**Background:**

Multiple prolonged symptoms are observed in patients who recover from acute coronavirus disease 2019 (COVID-19), defined as long COVID. Cough and sputum are presented by patients with long COVID during the acute and post-acute phases. This study aimed to identify specific risk factors for cough and sputum in patients with long COVID.

**Methods:**

Hospitalized patients with COVID-19 aged 18 years were enrolled in a multicenter cohort study at 26 medical institutions. Clinical data during hospitalization and patient-reported outcomes after discharge were collected from medical records, paper-based questionnaires, and smartphone apps.

**Results:**

At the 3-, 6-, and 12-month follow-ups, there were no differences in the incidence rates of wet and dry coughs. In contrast, the proportion of patients presenting sputum without coughing increased over time compared to those with sputum and coughing. Univariate analyses of cough and sputum at all follow-up visits identified intermittent mandatory ventilation (IMV), smoking, and older age as risk factors for prolonged symptoms. At the 12-month follow-up, persistent cough and sputum were associated with the characteristics of severe COVID-19 based on imaging findings, renal and liver dysfunction, pulmonary thromboembolism, and higher serum levels of LDH, KL-6, and HbA1C. The Kaplan–Meier curves showed that the severity of acute COVID-19 infection was correlated with prolonged cough and sputum production. Multivariable logistic regression analysis showed that IMV ventilator management were independent risk factors for prolonged cough and sputum at 12 months.

**Conclusions:**

In a Japanese population with long COVID, prolonged cough and sputum production were closely associated with severe COVID-19. These findings emphasize that a preventive approach including appropriate vaccination and contact precaution and further development of therapeutic drugs for COVID-19 are highly recommended for patients with risk factors for severe infection to avoid persistent respiratory symptoms.

**Supplementary Information:**

The online version contains supplementary material available at 10.1186/s12931-023-02591-3.

## Background

Coronavirus disease 2019 (COVID-19) is caused by the severe acute respiratory syndrome coronavirus 2 (SARS-CoV-2), which has spread worldwide since its outbreak in China in December 2019 [1, 2]. A large number of cases and deaths from COVID-19 have been reported, and the pandemic is ongoing, although vaccines are widely available. Most patients who have recovered from acute COVID-19 experience long-term effects on multiple organs and systems [3]. In most cases, persistent symptoms resolve over several months. However, some patients experience systemic symptoms that persist for more than 3–24 months. The World Health Organization (WHO) developed a consensus definition of the post-COVID-19 condition in 2021 [4]. To date, these sequelae have been called post-acute COVID-19, long-haul COVID, post-acute sequelae of SARS-CoV-2 infection (PASC), or post-acute syndrome of COVID-19. These systemic symptoms, including coughing and sputum, deteriorate the quality of life of patients and cause health and social burdens.

Cough is a protective response against the elimination of inhaled foreign substances and airway secretions [5]. Patients with acute or chronic respiratory diseases often present with cough. Persistent cough for > 8 weeks is defined as chronic cough accompanied by chronic respiratory diseases, including asthma, COPD, gastro-esophageal reflux disease, and otorhinolaryngological disorders with posterior nasal leakage [6]. Cough is classified as dry or productive, based on the presence or absence of sputum. Productive cough is primarily associated with pneumonia and/or bronchitis caused by infectious diseases [7]. Sputum is occasionally the main symptom without cough, especially in chronic respiratory diseases such as bronchiectasis [8]. Therapeutic strategies for cough and sputum are planned to treat underlying diseases using antitussive agents and expectorants. Cough during acute COVID-19 infection (acute COVID-19-associated cough) occurs in 40.5–72.5% of the patients. This symptom generally improves, and its incidence rate decreases to 18% at the 3-month follow-up [9]. Conversely, 10% of post-infected patients still have a prolonged cough 2 years after acute infection (post COVID-19 cough), while only 4% of COVID-19 uninfected patients exhibit cough [10].

Previous studies have reported that female sex, respiratory illness comorbidities, and severity of COVID-19 infection are risk factors for post-COVID-19 cough [9]. Unlike coughing following viral infections, including influenza, rhinovirus, and RS virus, post-COVID-19 cough is associated with various organ symptoms, such as fatigue and dyspnea, suggesting a different mechanism for this phenomenon [9]. In general, viral pneumonia mainly induces dry cough compared to bacterial pneumonia [11]. However, a previous study showed that the incidence rates of dry and productive cough in long-term COVID-19 patients were equal [12]. Additionally, the productive cough rate in patients with COVID-19 decreased from approximately 30–5% over 120 days after its onset [12, 13]. Nonetheless, the long-term natural history of cough and sputum in long COVID-19 remains unknown, and its longitudinal investigation of long COVID-19 is needed.

We conducted a longitudinal questionnaire survey on symptoms in COVID-19-infected patients over a 12-month period. We aimed to identify the specific risk factors for persistent cough and sputum in long COVID. This analysis focused on time-specific or temporal changes in these symptoms to provide a comprehensive evaluation.

## Methods

### Study design and participants

The detailed protocol for this project has been reported previously [14]. Briefly, prospective observational studies in Japan have been conducted in patients aged 18 years. The patients were admitted and discharged with a confirmed diagnosis of new coronavirus infection (COVID-19) (positive PCR or antigen test for SARS-CoV-2) from January 2020 until the end of February 2021 at 26 participating medical institutions. On 29 December 2020, we sent the study descriptions and consent forms to potential researchers from Keio University Hospital, the main study site. Subsequently, study invitations were mailed to 26 participating facilities that obtained approval from the Ethics Review Committee and permission to conduct the study.

The severity of COVID-19 during hospitalization was classified according to the guidelines of the Ministry of Health, Labor, and Welfare in Japan (Clinical management of patients with COVID-19: a guide for frontline healthcare workers, version 2.1.) as follows: mild, peripheral oxygen saturation (SpO2) ≥ 96%, with no respiratory symptoms or coughing only, no shortness of breath; moderate I (patient does not suffer respiratory failure), 93% < SpO2 < 96%, with shortness of breath and pneumonia findings; moderate II (patient suffers respiratory failure), SpO2 ≤ 93% and oxygen administration is required; severe, requiring admission to the intensive care unit (ICU) or mechanical ventilation.

Invitations to participate in the research were mailed to all inpatients within a certain period at each facility, and those who provided consent were asked to complete a questionnaire on paper or via a smartphone application at 3, 6, and 12 months after diagnosis.

### Questionnaire

The following symptoms were identified after COVID-19 diagnosis: fever, cough, sputum, breathlessness (dyspnea), sore throat, taste impairment, smell impairment, abdominal pain, and diarrhea. Other symptoms that were not present among the 24 representative symptoms described above could be filled in with an optional comment on the questionnaire. During hospitalization, patients were asked about the presence of each symptom at 3, 6, and 12 months after diagnosis.

Patients completed a paper-based questionnaire (paper patient-reported outcome [pPRO]) or a smartphone application-based questionnaire (electronic patient-reported outcome [ePRO]). Medical information covering 168 clinical survey items during medical treatment at each facility was collected over the Internet using an electronic data capture (EDC) system.

### Study population

**Fig. 1:**
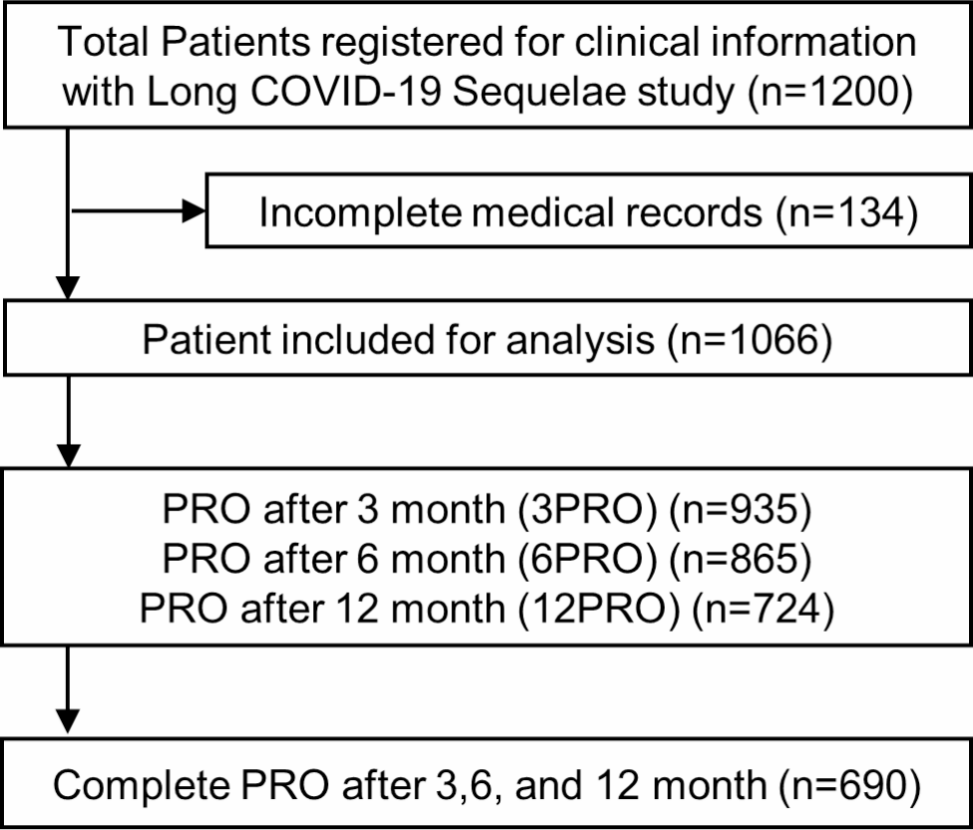
Flow chart of the study cohort. A total of 1200 patients were registered in the Long COVID-19 Sequelae study; 134 patients with incomplete medical records were excluded. A total of 1066 patients were included in the analysis. Patients reported questionnaires at 3-, 6-, and 12-months post-diagnosis. Among the patients, 935, 865, and 724 completed 3PRO, 6PRO, and 12PRO, respectively, and 690 completed all PROs. COVID-19, coronavirus disease 2019; PRO, patient-reported outcome

Figure 1 summarizes the patient population analyzed in this study based on the 3 (935 patients), 6 (865 patients), and 12 (724 patients) follow-up periods for the 1066 patients who answered the longitudinal questionnaire on symptoms at least once. To analyze temporal changes in the symptoms of long COVID, we analyzed the data of 690 patients who answered a longitudinal questionnaire on symptoms at all periods.

### Statistical analysis

Data are presented as mean and standard deviation (SD) or median (interquartile range (IQR)) for continuous variables and as number and percentage for categorical variables. We used the Student’s *t-*test and the chi-square test for group comparisons. Multivariable logistic regression was used to determine the independent predictors of persistent symptoms 12 months after recovery. Age and sex were entered into the multivariable logistic regression analysis, in addition to those that were significantly different in the univariate logistic regression analysis. All variables were included in the model. The log-rank test for trend and log-rank test were used to compare persistent symptoms according to COVID-19 severity. All analyses were performed with SPSS v.26 (IBM Corp., Armonk, NY, USA). A P value (two-tailed) of < 0.05 was considered statistically significant.

## Results

### Incidence of cough and sputum in acute and post-acute COVID-19 Infection during the 12-month follow-up

**Fig. 2:**
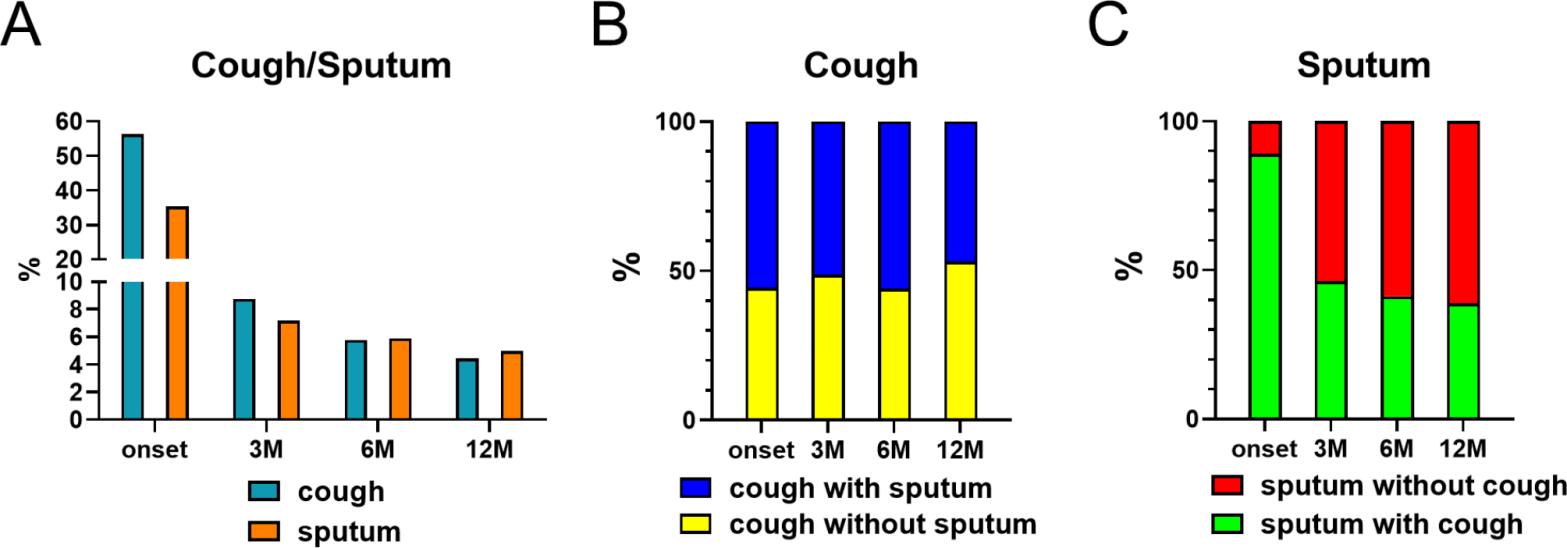
Assessment of prolonged cough and sputum thorough follow-up periods. (**A**) Prevalence of cough and sputum symptoms over time at each time point in patients who completed during follow-up. (**B**) Prevalence of cough with or without sputum (wet cough or dry cough, respectively) in patients who reported prolonged cough. **C**: Prevalence of sputum with or without cough in patients who reported prolonged sputum

The prevalence of cough and sputum decreased significantly from the acute phase to the 3-month follow-up, with only slight decreases at the 6- and 12-month follow-ups (cough: 52.3% at onset, 8.8% at 3 months, 5.8% at 6 months, and 4.4% at 12 months; sputum: 32.8% at onset, 7.2% at 3 months, 5.9% at 6 months, and 5.0% at 12 months) (Fig. 2A). Most patients with cough or sputum at the 12-month follow-up presented with these symptoms during hospitalization. At the 3-month follow-up, coughing was more frequent than sputum production. However, at the 6- and 12-month follow-ups, sputum was more frequent than cough. The incidence rates of dry cough and productive cough were similar at hospitalization and within the 12-month follow-up (55.7% at onset, 48.8% at 3 months, 56.0% at 6 months, and 46.9% at 12 months) (Fig. 2B). In contrast, sputum was accompanied by cough during hospitalization in almost all patients (89.0% at onset). At all follow-up periods, patients with sputum without cough were more frequent than those with cough, and the rate increased from the 3- to 12-month follow-up (62.7%, 54.9%, and 41.7%, at 3, 6 and 12 months respectively) **(**Fig. 2C**)**.

### Patient characteristics with prolonged cough in long COVID

Next, we compared patient characteristics between patients with and without cough at the 3-, 6-, and 12-month follow-ups (3-month: Supplementary Tables 1–3; 6-month: Supplementary Tables 5–7; 12-month: Tables 1, 2 and 3).

**Table 1:** Comorbidities, complications, and management of patients with and without cough or sputum in PRO after 12 months

	Cough			Sputum		
	Symptom (+) (N = 32)	Symptom (-) (N = 692)	*p* value	Symptom (+) (N = 36)	Symptom (-) (N = 688)	*p* value
Clinical characteristics						
Age, mean (95%CI)	64 (55.75–73.75)	59 (47–71)	0.038^a^	65 (53–75)	59 (47–70)	0.036^a^
Male, n (%)	24 (75)	422 (61)	0.111^b^	30 (83.3)	416 (60.5)	0.006^b^
BMI, mean (95%CI)	24.33 (22.14–27.71)	23.77 (21.45–26.26)	0.290^a^	23.77 (21.51–26.3)	24.14 (21.84–25.35)	0.917^a^
Current smoking, n (%)	4 (12.5)	70 (10.1)	0.693^b^	6 (16.7)	68 (10)	0.161^b^
Smoking history, n (%)	17 (53.1)	239 (34.5)	0.021^b^	18 (50)	238 (34.6)	0.042^b^
Comorbidities, n (%)						
Hypertension	19 (59.4)	238 (34.4)	0.004^b^	19 (52.8)	238 (34.6)	0.028^b^
Diabetes	14 (43.8)	108 (15.6)	< 0.001^b^	6 (16.7)	114 (16.6)	0.988^b^
Cardiovascular disease	3 (9.4)	40 (5.8)	0.409^b^	2 (5.6)	41 (59.6)	0.911^b^
Malignancy	2 (6.3)	40 (5.8)	0.920^b^	2 (5.6)	40 (58.1)	0.940^b^
COPD	2 (6.3)	22 (3.2)	0.350^b^	3 (8.3)	21 (3.1)	0.088^b^
Asthma	4(12.5)	36 (5.2)	0.082^b^	4 (11.1)	36 (5.2)	0.139^b^
Hyperuricemia	3 (9.4)	77 (11.1)	0.743^b^	7 (19.4)	73 (10.6)	0.105^b^
Chronic liver disorder	1 (3.1)	26 (3.8)	0.841^b^	1 (2.8)	26 (3.8)	0.745^b^
Chronic kidney disease	5 (15.6)	29 (4.2)	0.002^b^	2 (5.6)	32 (4.7)	0.789^b^
Management, n (%)						
ICU	7 (21.9)	65 (9.4)	0.024^b^	6 (16.7)	66 (9.6)	0.183
Mechanical ventilator	5 (15.6)	32 (4.6)	0.007^b^	6 (16.7)	31 (4.5)	0.002^b^
Use of IMV/NPPV/NHF	5 (15.6)	42 (6.1)	0.036^b^	6 (16.7)	41 (6.0)	0.013^b^
Abbreviation: PRO, patient reported outcome; BMI, body mass index; COPD, chronic obstructive pulmonary disease. ICU, intensive care unit.						

IMV/NPPV/NHF, intermittent mandatory ventilation/ noninvasive positive pressure ventilation/ nasal high flow. a t-test, b Chi-Square test.

**Table 2:** Clinical symptoms of patients with and without cough or sputum in PRO after 12 months

	Cough			Sputum		
	Symptom (+) (N = 32)	Symptom (-) (N = 692)	*p* value	Symptom (+) (N = 36)	Symptom (-) (N = 688)	*p* value
Clinical symptoms on admission, n (%)						
Fever	23 (71.9)	587 (84.8)	0.049^a^	29 (80.6)	581 (84.4)	0.532^a^
Cough	26 (81.3)	406 (58.7)	< 0.001^a^	27 (75.0)	405 (58.9)	0.054^a^
Sputum	22 (68.8)	248 (35.8)	< 0.001^a^	28 (77.8)	242 (35.2)	< 0.001^a^
Sore throat	12 (37.5)	194 (28.0)	0.189^a^	16 (44.4)	191 (27.7)	0.006^a^
Taste impairment	9 (28.1)	250 (36.1)	0.356^a^	16 (44.4)	215 (31.3)	0.098^a^
Smell impairment	6 (18.8)	235 (34.0)	0.074^a^	11 (30.6)	200 (29.1)	0.848^a^
Dyspnea	22 (68.8)	319 (46.1)	0.012^a^	22 (61.1)	315 (45.8)	0.072^a^
Abdominal pain	5 (15.6)	65 (9.4)	0.002^a^	5 (13.9)	65 (9.4)	0.379^a^
Diarrhea	4 (12.5)	151 (21.8)	0.209^a^	7 (19.4)	148 (21.5)	0.768^a^

^a^Chi-Square test.

**Table 3:** Laboratory and imaging findings of patients with and without cough or sputum in PRO after 12 months

	Cough			Sputum		
	Symptom (+) (N = 32)	Symptom (-) (N = 692)	*p* value	Symptom (+) (N = 36)	Symptom (-) (N = 688)	*p* value
Laboratory findings, median (IQR)						
WBC (cells/µl)	5070 (4125–7250)	4846 (3900–6200)	0.151^a^	5065 (4007-7457.5)	4840 (3900–6200)	0.121^a^
%NEU	73.5 (64.7–84.1)	68.95 (60–77)	0.004^a^	72 (63.2–82.8)	69 (60–77)	0.012^a^
%EOS	0 (0–1)	0 (0–1)	0.494^a^	0 (0–1)	0 (0–1)	0.099^a^
%LYM	17 (9.75-25)	22.5 (15.8–30)	0.006^a^	19 (10.3–26)	22.75 (15.3–30)	0.056^a^
Cre (mg/dl)	0.87 (0.65–1.08)	0.8 (0.65–0.97)	0.665^a^	0.86 (0.65-1.00)	0.81 (0.65–0.97)	0.947^a^
LDH (IU/L)	293 (239–402)	229 (190–297)	< 0.001^a^	278 (204–329)	230 (191-294.5)	0.087^a^
UA (mg/dl)	5 (4.05–5.85)	4.45 (3.575-5.5)	0.107^a^	5 (3.75–5.65)	4.5 (3.5–5.5)	0.245^a^
ferritin (ng/ml)	423 (171.75–639.9)	314 (150-596.9)	0.172^a^	409 (232-616.85)	314.5 (148.75-3597.18)	0.786^a^
KL-6 (U/ml)	304 (217–531)	220 (171–312)	0.022^a^	232 (177.75–336.5)	221.5 (172-318.75)	0.745^a^
HbA1c (%)	6.4 (5.9–7.2)	5.9 (5.6–6.4)	0.031^a^	6.05 (5.7–6.85)	5.9 (5.6–6.4)	0.539^a^
D-dimer (µg/ml)	0.9 (0.6–1.5)	0.8 (0.5–1.3)	0.576^a^	1.04 (0.7–1.55)	0.8 (0.5–1.3)	0.254^a^
Imaging examination n (%)						
Chest X-ray (GGO)	16 (50.0)	318 (46.0)	0.153^b^	22 (61.1)	312 (45.3)	0.179^b^
Chest X-ray (infiltration)	6 (18.8)	124 (17.9)	0.314^b^	8 (22.2)	122 (17.7)	0.198^b^
Chest X-ray ( ≧ 50% within 48 h)	2 (6.3)	49 (7.1)	0.848^b^	2 (6.3)	49 (71.2)	0.806^b^
Chest CT (GGO)	28 (87.5)	429 (62.0)	0.004^b^	31 (86.1)	426 (61.9)	0.128^b^
Chest CT (infiltration)	12 (37.5)	193 (27.9)	0.112^b^	13 (36.1)	192 (27.9)	0.169^b^

Abbreviation: WBC, white blood cell; NEU, neutrophil; EOS, eosinophil; LYM, lymphocyte; LDH, lactate dehydrogenase; CT, computed tomography; GGO, ground-glass opacity, ^a^ t-test, ^b^ Chi-Square test.

At the 3-month follow-up, the proportion of female sex was higher in the group of patients with a cough than in the group of patients without a cough (Supplementary Table 1). Multiple symptoms during the acute phase of COVID-19 during hospitalization were correlated with prolonged cough. Laboratory tests revealed higher levels of HbA1c in patients with cough. There were no significant differences in imaging findings between the two groups.

At the 6-month follow-up, a higher percentage of current smokers was found in patients with a cough than in patients without a cough (Supplementary Table 4). There was a positive correlation between cough and several other symptoms during hospitalization (Supplementary Table 5). Laboratory tests showed a higher ratio of neutrophils to blood leukocytes in patients with cough at the 6-month follow-up. Imaging findings demonstrated a higher proportion of patients in the cough group with chest radiographs showing consolidation and/or ground-glass opacity of more than 50% within 48 h after onset of acute COVID-19 infection patients with cough at this timepoint (Supplementary Table 6).

At the 12-month follow-up, a larger proportion of older patients presented cough group (Table 1). Patients with prolonged cough at this point presented several risk factors for severe COVID-19, including hypertension, diabetes, and chronic kidney disease (Table 1). The incidence of ICU admission, ventilator management, and renal dysfunction during hospitalization was significantly higher in the patients with cough (Table 1). Respiratory symptoms (cough, sputum, and dyspnea) and abdominal pain were correlated with prolonged cough at this point (Table 2). Laboratory tests revealed a higher ratio of neutrophils to peripheral blood leukocytes, a lower proportion of lymphocytes, and higher levels of LDH, KL-6, and HbA1C in patients with cough (Table 3). The imaging findings showed that a higher proportion of patients in the cough group at the 12-month follow-up had GGO on chest computed tomography (CT) images during hospitalization (Table 3).

### Patient characteristics with prolonged sputum in long COVID

Next, we compared patient characteristics between patients with sputum and those without sputum at the 3-, 6-, and 12-month follow-ups (3-month: Supplementary Tables 1–3; 6-month: Supplementary Tables 4–6; 12-month: Tables 1, 2 and 3).

At 3-month follow-up, different symptoms during hospitalization were correlated with prolonged sputum production (Supplementary Table 2). The incidence of ventilator management during hospitalization was significantly higher in patients with sputum production (Supplemental Table 1). Laboratory tests revealed a higher ratio of neutrophils to peripheral blood leukocytes and a lower proportion of lymphocytes in patients with prolonged sputum (Supplementary Table 3). There were no significant differences in the imaging findings between the two groups.

At the 6-month follow-up, multiple symptoms during hospitalization were correlated with sputum (Supplementary Table 5). The incidence of ventilator management during hospitalization was significantly higher in patients with sputum (Supplementary Table 4). Laboratory tests revealed a higher proportion of neutrophils in peripheral blood leukocytes, a lower proportion of lymphocytes, and higher LDH levels in patients with sputum (Supplementary Table 6). There were no significant differences in imaging findings between the two groups.

At the 12-month follow-up, a larger proportion of older patients presented sputum (Table 1). Male sex, smoking history, hypertension, and ventilator management during hospitalization were associated with prolonged sputum production (Table 1). Sputum production and sore throat during hospitalization correlated with prolonged sputum production (Table 2). Laboratory tests revealed a higher proportion of neutrophils in the peripheral blood leukocytes of patients with sputum (Table 3). There were no significant differences in imaging findings between the two groups.

### Risk factors for persistent cough and sputum in long COVID

At the 12-month follow-up, we identified multiple risk factors for persistent cough and sputum production that were closely associated with severe COVID-19.

**Fig. 3:**
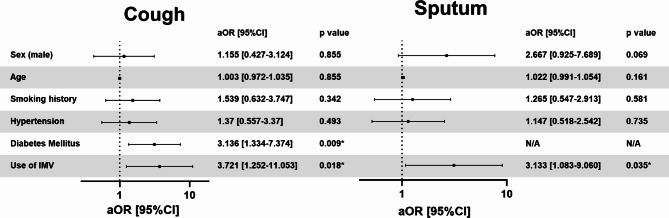
Multivariable logistic regression analysis for persistent cough and sputum at the 12-month follow-up. Multivariable logistic regression analysis for persistent cough and sputum at the 12-month follow-up was performed to demonstrate the independency among sex (male), age, smoking history, hypertension, diabetes mellitus, and use of IMV as the risk factor for persistent cough and sputum. Forest plot shows the point estimate and 95% confidence interval of adjusted odds ratio IMV, intermittent mandatory ventilation

First, we performed a univariate logistic regression analysis (Supplementary Table 7). Smoking history, hypertension, diabetes mellitus, and the need for intermittent mandatory ventilation (IMV) were common risk factors for prolonged coughing at the 12-month follow-up. Age, male sex, smoking history, and IMV use were common risk factors for prolonged sputum at the 12-month follow up. We performed a multivariable logistic regression analysis to evaluate age, sex, male sex, smoking history, hypertension, diabetes mellitus, and IMV use as potential risk factors for prolonged cough. We also performed a multivariable logistic regression analysis using age, sex, male sex, smoking history, hypertension, and IMV use as risk factors for prolonged sputum (Fig. 3). The use of IMV was an independent risk factor for prolonged coughing (adjusted odds ratio [aOR] 95% confidence interval [95%CI]: 3.721 [1.252–11.053], p = 0.018). Similarly, multivariable logistic regression analysis showed that the use of IMV was also an independent risk factor for prolonged sputum (aOR [95%CI] 3.078 [1.060–8.935, p = 0.039].

These findings indicate a positive correlation between ventilator management during hospitalization and persistent cough and sputum production in patients with prolonged COVID-19. In accordance with these findings, of the 690 patients who answered a longitudinal questionnaire on symptoms at all periods, 33 patients who received ventilator management during hospitalization had higher rates of cough symptoms and sputum at the 6-month (cough: 2.20 times; sputum: 2.60 times) and 12-month (cough: 2.57 times; sputum: 2.50 times) follow-up time points.

**Fig. 4:**
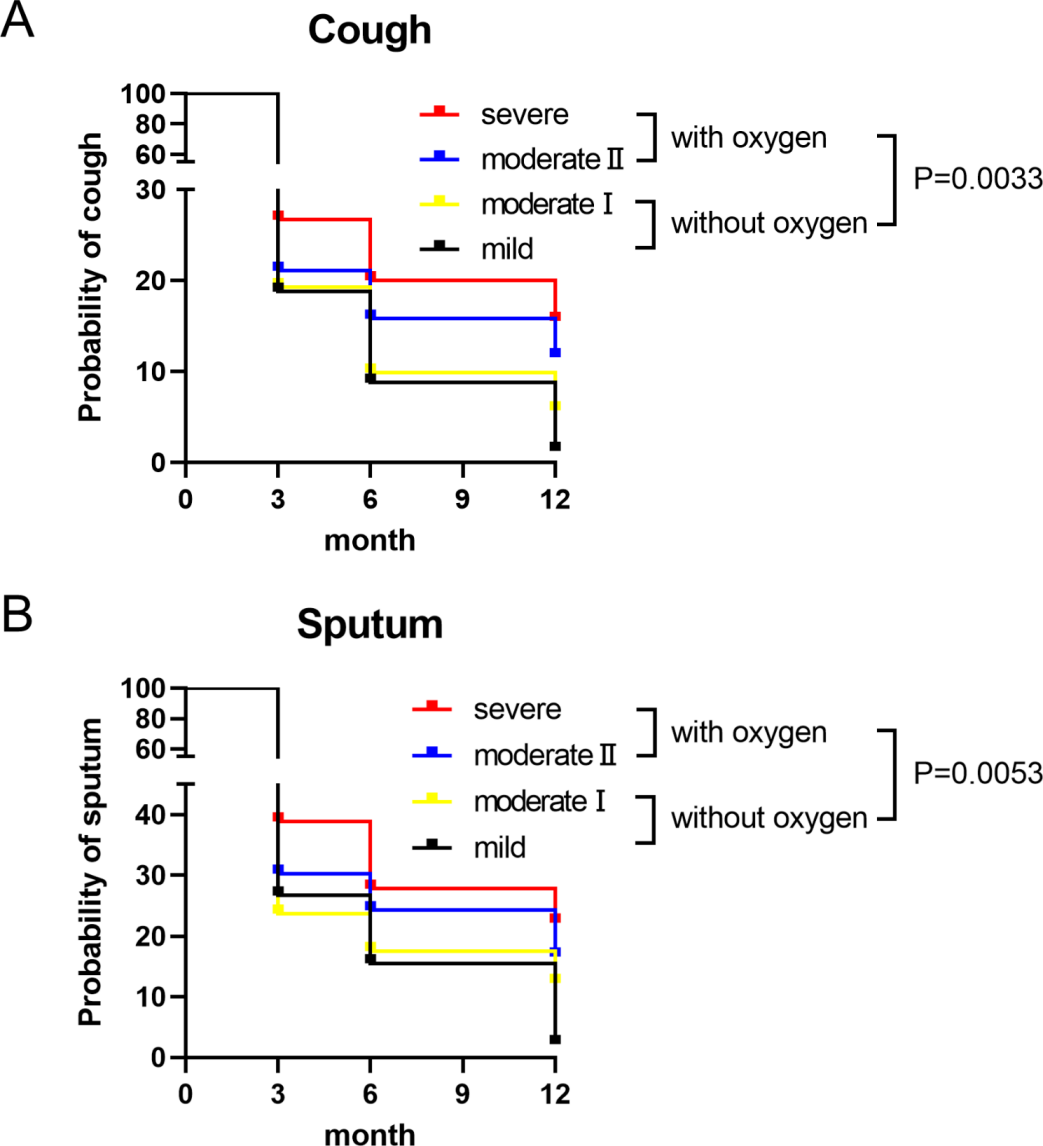
The relationship between prolonged cough and sputum during follow-up periods and the severity of acute COVID-19 infection. **A, B**) Longitudinal changes in both prolonged cough and sputum were analyzed using the Kaplan–Meier estimator in patients with different COVID-19 severity (mild, moderate I, moderate II and severe). **C, D**) Longitudinal changes in both prolonged cough and sputum were analyzed using the Kaplan–Meier estimator in patients with or without oxygen therapy during acute COVID-19 infection. Group 1: Patients without oxygen therapy. Group 2: Patients who needed oxygen therapy

Patients who received ventilator management during hospitalization were defined as having severe acute COVID-19. Thus, we analyzed the relationship between prolonged cough and follow-up periods using classification of severity in acute COVID-19 infection: moderate II and severe COVID-19 patients who required oxygen therapy more frequently presented cough at the 12-month follow-up than non-severe patients who did not require oxygen therapy (Fig. 4A: Log-rank (Mantel-Cox) test: p = 0.0033, HR [95%CI] 1.843 [1.225–2.771]). In addition, patients who required oxygen therapy more frequently presented sputum at the 12-month follow-up than non-severe patients (Fig. 4B: Log-rank (Mantel Cox) test: p = 0.0053, HR [95%CI] 1.875 [1.205–2.916]).

## Discussion

This study was a longitudinal evaluation of cough and sputum production during the first 12 months after acute COVID-19 infection. To our knowledge, this is the first detailed report of sputum in patients with long-term COVID. Although the frequency of dry and productive cough was similar, the number of patients with sputum not accompanied by cough increased at 6 and 12 months after acute COVID-19 infection. This study identified the relationship between cough and sputum in long COVID and risk factors for severe infection. Among these, ventilator management was especially correlated with cough and sputum production during prolonged COVID. Furthermore, the severity of COVID-19 tended to be related to prolonged coughing and sputum formation during follow-up.

In severe cases of COVID-19, pneumonia progression causes respiratory failure due to tissue damage [15]. Airway inflammation with epithelial damage has also been observed during the acute phase of COVID-19 infection. A previous study has shown that COVID-19 leads to pulmonary fibrosis and bronchiectasis [3]. In general, severe pneumonia increases cough sensitivity and secretions from proliferating goblet cells. These changes are particularly evident following long-term ventilator management. In this study, correlations with severity in the ratio of prolonged cough to sputum were observed, suggesting a relationship between pneumonia and these symptoms in long COVID. Importantly, the pathogenic mechanism might differ between the symptoms during the acute phase and those after recovery because the former is induced partly by upper airway inflammation.

In this study, cough was correlated with the severity and risk factors of severe COVID-19, especially at the 12-month follow-up. Cough is frequently observed as a respiratory symptom in both upper respiratory infections and pneumonia [5]. Post-infectious cough due to respiratory viral infection is the most common cause of chronic cough that occasionally persists for up to three months [16]. Our analysis showed that cough at the 3- and 6-month follow-ups was correlated with pharyngeal pain during hospitalization. In contrast, cough at the 12-month follow-up showed no correlation, suggesting that persistent cough after 6 months may primarily depend on lung damage and not on upper airway inflammation [17].

Several previous reports have documented the prevalence of cough over time; Arnold et al. reported that 60–70% of patients complained of cough at admission, which decreased to approximately 10% at 12 weeks after admission due to COVID-19 infection [18]. Carfi et al. reported that cough in the acute phase was present in approximately 70% of patients and had decreased to approximately 15% after an average of 60 days of acute COVID-19 infection [19]. However, D’Cruz et al. reported that prolonged coughing was present in 42.6% of the patients up to six months later [20]. Prevalence over time is expected to vary greatly depending on whether the patient population under analysis includes inpatients or outpatients. All patients included in our cohort were hospitalized, and the residual cough rate tended to be higher at the time of the study than in previous studies that used outpatient information.

In this study, the prolonged symptoms of inpatients with acute COVID-19 infection were analyzed to evaluate long-term COVID, and the rate of patients with prolonged sputum was higher than that observed in previous studies that mainly relied on outpatient information [13]. The higher incidence of prolonged sputum culture enabled us to analyze the risk factors in this study. We found that sputum was correlated with older age, male sex, and smoking history only at the 12-month follow-up visit. In contrast, ventilator management in patients with severe COVID-19 consistently correlated with sputum during at the 12-month follow-up. In general, the incidence rate of sputum is higher in older patients and current smokers [21]. Goblet cell hyperplasia is often observed in chronic airway inflammatory diseases such as asthma and COPD [22]. Similarly, persistent airway inflammation caused by severe COVID-19 may promote goblet cell hyperplasia and long-term mucus production. Furthermore, although a significantly increased frequency of cough in patients with obstructive lung disease has also been reported in another long COVID study [20], there was no difference in the prevalence of asthma or COPD in patients with prolonged cough throughout the follow-up period.

Dry cough is common in viral pneumonia, suggesting that specific risk factors may be necessary for persistent sputum formation after viral pneumonia with regard to sputum [11]. Bronchiolitis secondary to bacterial infections is the most common cause of purulent sputum after viral infections. Although a higher rate of secondary bacterial infections and higher mortality in COVID-19 infection has been reported than in influenza infection [10], there was no significant difference in the ratio of bacterial infection between the groups with and without cough in this study. Secondary bacterial infections may be underdiagnosed in the clinical setting.

This study had several limitations. First, not all enrolled patients answered all symptom questionnaires at the 3-, 6-, and 12-month follow-ups. Therefore, the overall population differed according to the analysis. Second, pulmonary function tests, or imaging studies, and the 6-minute walk test were not performed after acute COVID-19 infection. Therefore, the causal relationship after cough-, sputum-, and pneumonia-induced tissue damage remains unknown. Third, the degree of symptoms was not quantified, which may have rendered the evaluation of causal relationships inadequate.

## Conclusions

This study clarified the frequency and characteristics of coughing and sputum in long COVID. Importantly, both symptoms were highly associated with the severity and risk factors of severe COVID-19. Therefore, it is necessary to recognize that severe infections can cause sequelae of cough and sputum in clinical settings. In addition, these findings emphasize that a preventive approach including appropriate vaccination and contact precaution and further development of therapeutic drugs for COVID-19 should be highly recommended for patients with risk factors for severe infection. Long-term persistent cough and sputum are burdens on patients after recovery from acute COVID-19. Further investigations are needed to elucidate the underlying mechanisms and develop specific therapeutic drugs for these persistent symptoms in patients with long COVID.

### Electronic supplementary material

Below is the link to the electronic supplementary material.

Supplementary Material 1

## Data Availability

The datasets used and/or analyzed during the current study are available from the corresponding author on reasonable request.

## Abbreviations

COVID-19: Coronavirus disease 2019
ICU: Intensive care unit
IMV: Intermittent mandatory ventilation
PASC: Post-acute sequelae of SARS-CoV-2 infection
PRO: Patient-reported outcome
SpO2: Peripheral oxygen saturation
