# Supplementary material for: Cough and sputum in long COVID are associated with severe acute COVID-19: a Japanese cohort study
Source: Respir Res. 2023 Nov 14;24:283. doi: 10.1186/s12931-023-02591-3 (PMC10648313; doi:10.1186/s12931-023-02591-3)
Supplement: Supplementary file 1 — Supplementary Material 1 [file 12931_2023_2591_MOESM1_ESM.docx]

| Supplementary table 1. Comorbidities, complications, and management of patients with and without cough or sputum in PRO after 3 months. | | | | | | |
| --- | --- | --- | --- | --- | --- | --- |
|  | Cough | | | Sputum | | |
|  | Symptom (+) (N=82) | Symptom (-) (N=852) | *p* value | Symptom (+) (N=67) | Symptom (-) (N=868) | *p* value |
| Clinical characteristics | | | | | | |
| Age, mean (95%CI) | 56.5 (28-83.9) | 56.9 (26-81) | 0.803^a^ | 60.18 (32.8-82.6) | 56.62 (26-81) | 0.088^a^ |
| Male, n (%) | 42 (51.2) | 552 (64.8) | 0.015^b^ | 46 (68.6) | 549 (63.2) | 0.375^b^ |
| BMI, mean (95%CI) | 24.2 (24.2-27.4) | 23.8 (21.5-26.3) | 0.845^a^ | 24.6 (19-31.8) | 24.1 (18.2-31.9) | 0.449^a^ |
| Current smoking, n (%) | 8 (9.8) | 93 (10.9) | 0.674^b^ | 6 (9) | 96 (11.1) | 0.161^b^ |
| Smoking history, n (%) | 24 (2.9) | 47 (5.5) | 0.192^b^ | 22 (32.8) | 306 (35.3) | 0.042^b^ |
| Comorbidities, n (%) | | | | | | |
| Hypertension | 26 (31.7) | 290 (34) | 0.693^b^ | 29 (43.3) | 287 (33.1) | 0.098^b^ |
| Diabetes | 10 (12.2) | 147 (17.3) | 0.244^b^ | 11 (16.4) | 146 (16.8) | 0.900^b^ |
| Cardiovascular disease | 5 (6.1) | 54 (6.3) | 0.963^b^ | 2 (3) | 57 (6.6) | 0.250^b^ |
| Malignancy | 7 (8.5) | 52 (6.1) | 0.364^b^ | 3 (4.5) | 56 (6.5) | 0.529^b^ |
| COPD | 4 (4.9) | 27 (3.2) | 0.407^b^ | 4 (6) | 27 (3.1) | 0.216^b^ |
| Asthma | 6 (7.3) | 45 (5.3) | 0.424^b^ | 5 (7.5) | 46 (5.3) | 0.452^b^ |
| Hyperuricemia | 3 (3.7) | 96 (11.3) | 0.033^b^ | 5 (7.5) | 94 (10.8) | 0.375^b^ |
| Chronic liver disorder | 3 (3.7) | 30 (3.5) | 0.956^b^ | 1 (1.5) | 32 (3.7) | 0.338^b^ |
| Chronic kidney disease | 1 (1.2) | 41 (4.8) | 0.136^b^ | 2 (3) | 40 (4.6) | 0.518^b^ |
| Management, n (%) | | | | | | |
| ICU | 8 (9.8) | 85 (10) | 0.934^b^ | 10 (14.9) | 83 (9.6) | 0.177^b^ |
| Mechanical ventilator | 7 (8.5) | 36 (4.2) | 0.079^b^ | 7 (10.4) | 36 (4.1) | 0.021^b^ |
| Use of IMV/NPPV/NHF | 9 (11) | 72 (8.5) | 0.051^b^ | 8 (11.9) | 47 (5.4) | 0.054^b^ |
| Abbreviation: PRO, patient reported outcome; BMI, body mass index; COPD, chronic obstructive pulmonary disease; ICU, intensive care unit. | | | | | | |
| IMV/NPPV/NHF, intermittent mandatory ventilation/ noninvasive positive pressure ventilation/ nasal high flow. ^a^t-test, ^b^Chi-Square test. | | | | | | |

| Supplementary Table 2. Clinical symptoms of patients with and without cough or sputum in PRO after 3 months. | | | | | | |
| --- | --- | --- | --- | --- | --- | --- |
|  | Cough | | | Sputum | | |
|  | Symptom (+) (N=82) | Symptom (-) (N=852) | *p* value | Symptom (+) (N=67) | Symptom (-) (N=868) | *p* value |
| Clinical symptoms on admission, n (%) | | | | | | |
| Fever | 65 (79.3) | 726 (85.2) | <0.001^a^ | 56 (83.6) | 736 (84.8) | 0.312^a^ |
| Cough | 79 (96.3) | 484 (56.8) | <0.001^a^ | 59 (88.1) | 505 (58.2) | <0.001^a^ |
| Sputum | 56 (68.3) | 296 (34.7) | <0.001^a^ | 60 (89.6) | 293 (33.8) | <0.001^a^ |
| Sore throat | 33 (40.2) | 236 (27.7) | <0.001^a^ | 39 (58.2) | 231 (26.6) | <0.001^a^ |
| Taste impairment | 36 (43.9) | 299 (35.1) | 0.249^a^ | 30 (44.8) | 273 (31.5) | 0.025^a^ |
| Smell impairment | 35 (42.7) | 277 (32.5) | 0.031^a^ | 25 (37.3) | 250 (28.8) | 0.141^a^ |
| Dyspnea | 57 (69.5) | 386 (45.3) | <0.001^a^ | 49 (73.1) | 395 (45.5) | <0.001^a^ |
| Abdominal pain | 14 (17.1) | 80 (9.4) | <0.001^a^ | 16 (23.9) | 78 (9.0) | <0.001^a^ |
| Diarrhea | 26 (31.7) | 183 (21.5) | <0.001^a^ | 28 (41.8) | 181 (20.9) | <0.001^a^ |
| ^a^Chi-Square test. | | | | | | |

| Supplementary table 3. Laboratory and imaging findings of patients with and without cough or sputum in PRO after 3 months. | | | | | | |
| --- | --- | --- | --- | --- | --- | --- |
|  | Cough | |  | Sputum | |  |
|  | Symptom (+) (N=82) | Symptom (-) (N=852) | *p* value | Symptom (+) (N=67) | Symptom (-) (N=868) | *p* value |
| Laboratory findings, median (IQR) | | | | | | |
| WBC (cells/μl) | 5000 (3885-6575) | 4800 (3900-6100) | 0.447^a^ | 5200 (4200-6340) | 4765 (3860-6100) | 0.010^a^ |
| %NEU | 68.9 (61.4-80.1) | 68.0 (60.0-76.6) | 0.490^a^ | 72 (62.1-81) | 67.3 (59.8-76) | 0.010^a^ |
| %EOS | 0.2 (0-1) | 0 (0-1) | 0.673^a^ | 0 (0-1) | 0.1 (0-1) | 0.594^a^ |
| %LYM | 22.2 (11.6-28.9) | 23 (16-30) | 0.380^a^ | 19.5 (5.6-43.3) | 24.0 (8-42) | 0.015^a^ |
| Cre (mg/dl) | 0.78 (0.64-0.92) | 0.82 (0.68-0.98) | 0.578^a^ | 0.82 (0.66-0.95) | 0.81 (0.51-1.37) | 0.578^a^ |
| LDH (IU/L) | 238 (199-310) | 230 (188-297) | 0.932^a^ | 248 (208-329.5) | 230 (188-293.75) | 0.068^a^ |
| UA (mg/dl) | 4.7 (3.8-5.7) | 4.5 (3.6-5.5) | 0.102^a^ | 4.8 (3.7-5.7) | 4.5 (3.6-5.5) | 0.117^a^ |
| Ferritin (ng/ml) | 270 (84.3-508.6) | 335 (167.5-621.5) | 0.191^a^ | 378 (195-612.7) | 321.5 (155.8-613.8) | 0.775^a^ |
| KL-6 (U/ml) | 250.5 (171.5-380.8) | 220 (128-585.5) | 0.702^a^ | 205 (170.75-410.25) | 222.5 (176-310.25) | 0.530^a^ |
| HbA1c (%) | 6 (5.6-6.2) | 5.9 (5.6-6.5) | 0.019^a^ | 6.0 (5.6-6.3) | 5.9 (5.6-6.4) | 0.644^a^ |
| D-dimer (μg/ml) | 0.9 (0.6-1.35) | 0.9 (0.5-1.3) | 0.283^a^ | 0.9 (0.6-1.3) | 0.8 (0.5-1.3) | 0.363^a^ |
| Imaging examination, n (%) | | | | | | |
| Chest X-ray (GGO) | 33 (40.2) | 389 (45.7) | 0.387^b^ | 34 (5.1) | 391 (45) | 0.589^b^ |
| Chest X-ray (infiltration) | 11 (13.4) | 148 (17.4) | 0.665^b^ | 9 (13.4) | 150 (17.3) | 0.624^b^ |
| Chest X-ray (≧50% within 48hrs) | 6 (7.3) | 56 (6.6) | 0.126^b^ | 4 (6) | 58 (6.7) | 0.970^b^ |
| Chest CT (GGO) | 57 (69.5) | 531 (62.3) | 0.370^b^ | 49 (73.1) | 540 (62.2) | 0.822^b^ |
| Chest CT (infiltration) | 16 (19.5) | 238 (27.9) | 0.296^b^ | 17 (25.4) | 237 (27.3) | 0.779^b^ |
| Abbreviation: WBC, white blood cell; NEU, neutrophil; EOS, eosinophil; LYM, lymphocyte; Cre, creatinine; LDH, lactate dehydrogenase;  CT, computed tomography; GGO, ground-glass opacity; ^a^t-test, ^b^Chi-Square test. | | | | | | |

| Supplementary table 4. Comorbidities, complications, and management of patients with and without cough or sputum in PRO after 6 months. | | | | | | |
| --- | --- | --- | --- | --- | --- | --- |
|  | Cough | | | Sputum | | |
|  | Symptom (+) (N=50) | Symptom (-) (N=815) | *p* value | Symptom (+) (N=51) | Symptom (-) (N=814) | *p* value |
| Clinical characteristics | | | | | | |
| Age, mean (95%CI) | 56.8 (33.1-81.5) | 56.8 (26.8-81.2) | 0.995^a^ | 61.1 (35-81.4) | 56.5 (26.8-81.3) | 0.088^a^ |
| Male, n (%) | 28 (56) | 522 (64) | 0.251^b^ | 46 (68.6) | 549 (63.2) | 0.375^b^ |
| BMI, mean (95%CI) | 24.0 (2.0-27.5) | 24.2 (18.2-31.9) | 0.887^a^ | 23.6 (17.9-30.4) | 24.2 (18.3-32) | 0.833^a^ |
| Current smoking, n (%) | 24.1 (18.3-31.6) | 81 (9.9) | 0.039^b^ | 6 (9) | 96 (11.1) | 0.161^b^ |
| Smoking history, n (%) | 18 (36) | 286 (35.1) | 0.950^b^ | 22 (32.8) | 306 (35.3) | 0.042^b^ |
| Comorbidities, n (%) | | | | | | |
| Hypertension | 18 (36) | 280 (34.4) | 0.836^b^ | 23 (45.1) | 275 (33.8) | 0.106^b^ |
| Diabetes | 7 (14) | 137 (16.8) | 0.582^b^ | 7 (13.7) | 137 (16.8) | 0.542^b^ |
| Cardiovascular disease | 1 (2) | 51 (6.3) | 0.215^b^ | 0 (0) | 52 (6.4) | 0.061^b^ |
| Malignancy | 2 (4) | 47 (5.8) | 0.590^b^ | 1 (2) | 48 (5.9) | 0.233^b^ |
| COPD | 4 (8) | 24 (2.9) | 0.052^b^ | 4 (7.8) | 24 (2.9) | 0.068^b^ |
| Asthma | 4 (8) | 41 (5) | 0.350^b^ | 2 (3.9) | 43 (5.3) | 0.679^b^ |
| Hyperuricemia | 3 (6) | 88 (10.8) | 0.276^b^ | 5 (9.8) | 86 (10.6) | 0.848^b^ |
| Chronic liver disorder | 3 (6) | 28 (3.4) | 0.258^b^ | 1 (2) | 30 (3.7) | 0.509^b^ |
| Chronic kidney disease | 0 (0) | 41 (5) | 0.105^b^ | 1 (2) | 40 (4.9) | 0.338^b^ |
| Management, n (%) | | | | | | |
| ICU | 4 (8) | 77 (9.4) | 0.737^b^ | 7 (13.7) | 74 (9.1) | 0.220^b^ |
| Mechanical ventilator | 4 (8) | 31 (3.8) | 0.143^b^ | 7 (13.7) | 28 (3.4) | <0.001^b^ |
| Use of IMV/NPPV/NHF | 4 (8) | 31 (3.8) | 0.137^b^ | 7 (!3.7) | 28 (3.4) | <0.001^b^ |
| Abbreviation: PRO, patient reported outcome; BMI, body mass index; COPD, chronic obstructive pulmonary disease; ICU, intensive care unit; | | | | | | |
| IMV/NPPV/NHF, intermittent mandatory ventilation/ noninvasive positive pressure ventilation/ nasal high flow. ^a^t-test, ^b^Chi-Square test. | | | | | | |

| Supplementary table 5. Clinical symptoms of patients with and without cough or sputum in PRO after 6 months. | | | | | | |
| --- | --- | --- | --- | --- | --- | --- |
|  | Cough | | | Sputum | | |
|  | Symptom (+) (N=50) | Symptom (-) (N=815) | *p* value | Symptom (+) (N=51) | Symptom (-) (N=814) | *p* value |
| Clinical symptoms on admission, n (%) | | | | | | |
| Fever | 43 (86) | 726 (89.1) | 0.079^a^ | 41 (80.4) | 689 (84.6) | 0.417^a^ |
| Cough | 43 (86) | 480 (58.9) | <0.001^a^ | 44 (86.3) | 479 (58.8) | <0.001^a^ |
| Sputum | 35 (70) | 284 (34.8) | <0.001^a^ | 45 (88.2) | 274 (33.7) | <0.001^a^ |
| Sore throat | 24 (48) | 218 (26.7) | <0.001^a^ | 31 (60.8) | 211 (25.9) | <0.001^a^ |
| Taste impairment | 21 (42) | 287 (35.2) | 0.208^a^ | 24 (47.1) | 254 (31.2) | 0.019^a^ |
| Smell impairment | 19 (38) | 273 (33.5) | 0.269^a^ | 16 (31.4) | 241 (29.6) | 0.789^a^ |
| Dyspnea | 32 (64) | 372 (45.6) | <0.001^a^ | 35 (68.6) | 369 (45.3) | 0.001^a^ |
| Abdominal pain | 10 (20) | 77 (9.4) | 0.002^a^ | 10 (19.6) | 87 (10.7) | 0.050^a^ |
| Diarrhea | 18 (36) | 172 (21.1) | <0.001^a^ | 16 (31.4) | 174 (21.4) | 0.094^a^ |
| ^a^Chi-Square test. | | | | | | |

| Supplementary table 6. Laboratory and imaging findings of patients with and without cough or sputum in PRO after 6 months. | | | | | | |
| --- | --- | --- | --- | --- | --- | --- |
|  | Cough | |  | Sputum | |  |
|  | Symptom (+) (N=50) | Symptom (-) (N=815) | *p* value | Symptom (+) (N=51) | Symptom (-) (N=814) | *p* value |
| Laboratory findings, median (IQR) | | | | | | |
| WBC (cells/μl) | 5000 (2415-9665) | 4800 (3900-6130) | 0.798^a^ | 5000 (3765-6060) | 4800 (3900-6130) | 0.010^a^ |
| %NEU | 71 (54.4-89.9) | 67.6 (59.7-76.6) | 0.034^a^ | 71 (63.5-80.0) | 67.6 (59.7-76.6) | 0.013^a^ |
| %EOS | 0.2 (0-3.6) | 0.05 (0-1) | 0.957^a^ | 0.2 (0-1) | 0.5 (0-1) | 0.208^a^ |
| %LYM | 22 (6-35.2) | 23 (16-30) | 0.160^a^ | 22 (13-27) | 23 (16-30) | 0.042^a^ |
| Cre (mg/dl) | 0.74 (0.5-1.06) | 0.82 (0.66-0.98) | 0.606^a^ | 0.74 (0.6-0.88) | 0.82 (0.66-0.98) | 0.643^a^ |
| LDH (IU/L) | 254 (154.8-460.4) | 227 (188-295) | 0.110^a^ | 264.5 (137.3-493.6) | 227 (188-295) | 0.038^a^ |
| UA (mg/dl) | 4.4 (2.6-9.2) | 4.5 (3.6-5.5) | 0.796^a^ | 4.4 (3.3-5.9) | 4.5 (3.6-5.6) | 0.111^a^ |
| Ferritin (ng/ml) | 264.5 (23.8-1152.7) | 317 (162.3-616) | 0.014^a^ | 264.5 (137.3-493.6) | 317.3 (162.3-616) | 0.951^a^ |
| KL-6 (U/ml) | 242.5 (176-387.8) | 220 (172-311.8) | 0.790^a^ | 242.5 (176-387.8) | 220 (172-311.75) | 0.395^a^ |
| HbA1c (%) | 6 (5.8-6.4) | 5.9 (5.6-6.4) | 0.962^a^ | 6.0 (5.8-6.4) | 5.9 (5.6-6.4) | 0.727^a^ |
| D-dimer (μg/ml) | 0.85 (0.5-1.5) | 0.8 (0.5-1.3) | 0.439^a^ | 0.85 (0.5-1.5) | 0.8 (0.5-1.3) | 0.559^a^ |
| Imaging examination, n (%) | | | | | | |
| Chest X-ray (GGO) | 18 (38.3) | 372 (45.6) | 0.068^b^ | 24 (47.1) | 366 (45) | 0.931^b^ |
| Chest X-ray (infiltration) | 6 (12.8) | 137 (16.8) | 0.260^b^ | 8 (15.7) | 135 (16.6) | 0.825^b^ |
| Chest X-ray (≧50% within 48hrs) | 2 (4.3) | 56 (6.8) | 0.013^b^ | 2 (3.9) | 56 (6.9) | 0.645^b^ |
| Chest CT (GGO) | 5 (10.6) | 506 (62.1) | 0.312^b^ | 38 (74.5) | 505 (62) | 0.298^b^ |
| Chest CT (infiltration) | 10 (21.3) | 225 (27.6) | 0.118^b^ | 12 (23.5) | 223 (27.4) | 0.800^b^ |
| Abbreviation: WBC, white blood cell; NEU, neutrophil; EOS, eosinophil; LYM, lymphocyte; Cre, creatinine; LDH, lactate dehydrogenase;  CT, computed tomography; GGO, ground-glass opacity. ^a^t-test, ^b^Chi-Square test. | | | | | | |

| Supplementary table 7. Univariate analysis for persistent cough and sputum at 12 months. | | | | | | |
| --- | --- | --- | --- | --- | --- | --- |
|  |  | Cough |  |  | Sputum |  |
|  | OR | 95%CI | *p* value | OR | 95%CI | *p* value |
| **Clinical characteristics** | | | | | | |
| Sex (male) | 1.919 | 0.850-4.335 | 0.117 | 3.269 | 1.343-7.959 | 0.009 |
| Age | 1.021 | 0.997 | 1.046 | 1.025 | 1.001-1.049 | 0.037 |
| BMI | 1.048 | 0.962-1.141 | 0.285 | 1.004 | 0.926-1.089 | 0.917 |
| Smoking history | 2.178 | 1.002-4.732 | 0.049 | 2.116 | 1.001-4.475 | 0.050 |
| **Comorbidities** | | | | | | |
| Hypertension | 2.763 | 1.341-5.693 | 0.006 | 2.094 | 1.069-4.105 | 0.031 |
| Diabetes | 4.241 | 2.047-8.787 | <0.001 | 0.993 | 0.404-2.441 | 0.988 |
| Cardiovascular disease | 1.671 | 0.488-5.721 | 0.414 | 0.92 | 0.213-3.962 | 0.911 |
| Malignancy | 1.078 | 0.249-4.674 | 0.920 | 0.946 | 0.219-4.077 | 0.940 |
| COPD | 2.009 | 0.451-8.942 | 0.360 | 2.857 | 0.811-10.065 | 0.102 |
| Asthma | 2.567 | 0.855-7.714 | 0.093 | 2.233 | 0.749-6.656 | 0.150 |
| Hyperuricemia | 0.817 | 0.243-2.745 | 0.744 | 2.01 | 0.85-4.752 | 0.112 |
| Chronic liver disorder | 0.813 | 0.107-6.185 | 0.841 | 0.715 | 0.094-5.428 | 0.746 |
| Chronic kidney disease | 4.324 | 1.548-12.072 | 4.324 | 1.222 | 0.281-5.317 | 0.790 |
| **Management** | | | | | | |
| ICU | 2.641 | 1.099-6.342 | 0.030 | 1.842 | 0.74-4.589 | 0.189 |
| Oxygen demand above nasal canula | 2.852 | 1.392-5.842 | 0.004 | 1.954 | 0.996-3.835 | 0.051 |
| Use of IMV | 3.733 | 1.349-10.332 | 0.011 | 4.142 | 1.605-10.686 | 0.003 |
| Abbreviation: BMI, body mass index; COPD, chronic obstructive pulmonary disease; GGO, ground-glass opacity; ICU, intensive care unit; | | | | | | |
| IMV, intermittent mandatory ventilation | | | | | | |
